# Oxytocin and Fear Memory Extinction: Possible Implications for the Therapy of Fear Disorders?

**DOI:** 10.3390/ijms221810000

**Published:** 2021-09-16

**Authors:** Elisabetta Baldi, Alessia Costa, Barbara Rani, Maria Beatrice Passani, Patrizio Blandina, Adele Romano, Gustavo Provensi

**Affiliations:** 1 Section of Physiological Sciences, Department of Experimental and Clinical Medicine, University of Florence, 50134 Florence, Italy; e.baldi@unifi.it; 2 Section of Clinical Pharmacology and Oncology, Department of Health Sciences (DSS), University of Florence, 50139 Florence, Italy; alessia.costa@unifi.it (A.C.); barbara.rani@unifi.it (B.R.); beatrice.passani@unifi.it (M.B.P.); 3 Section of Pharmacology of Toxicology, Department of Neuroscience, Psychology, Drug Research and Child Health (NEUROFARBA), University of Florence, 50139 Florence, Italy; patrizio.blandina@unifi.it; 4 Department of Physiology and Pharmacology ‘V. Erspamer’, Sapienza University of Rome, 00185 Rome, Italy; adele.romano@uniroma1.it

**Keywords:** oxytocin, fear extinction, exposure therapy, amygdala, medial prefrontal cortex, infralimbic cortex, prelimbic cortex, hippocampus

## Abstract

Several psychiatric conditions such as phobias, generalized anxiety, and post-traumatic stress disorder (PTSD) are characterized by pathological fear and anxiety. The main therapeutic approach used in the management of these disorders is exposure-based therapy, which is conceptually based upon fear extinction with the formation of a new safe memory association, allowing the reduction in behavioral conditioned fear responses. Nevertheless, this approach is only partially resolutive, since many patients have difficulty following the demanding and long process, and relapses are frequently observed over time. One strategy to improve the efficacy of the cognitive therapy is the combination with pharmacological agents. Therefore, the identification of compounds able to strengthen the formation and persistence of the inhibitory associations is a key goal. Recently, growing interest has been aroused by the neuropeptide oxytocin (OXT), which has been shown to have anxiolytic effects. Furthermore, OXT receptors and binding sites have been found in the critical brain structures involved in fear extinction. In this review, the recent literature addressing the complex effects of OXT on fear extinction at preclinical and clinical levels is discussed. These studies suggest that the OXT roles in fear behavior are due to its local effects in several brain regions, most notably, distinct amygdaloid regions.

## 1. Introduction

Pathological fear and anxiety are a hallmark of many psychiatric conditions, such as phobias, generalized anxiety, and post-traumatic stress disorder (PTSD). For example, PTSD patients show strong traumatic memories that are retrieved in an intrusive manner, causing re-experiencing of the traumatic event and increased arousal and stress response. The persistence of PTSD can be explained in terms of trauma-induced strengthening of the fear memory or failure to extinguish it [1,2]. These disorders not only seriously undermine the mental health of the affected individuals, but also raise an enormous public health and economic burden on society [3]. For this reason, considerable efforts have been made in the last decades to understand the neurobiological and psychological mechanisms underlying these disorders [4,5]. As most of the mentioned disorders are characterized by pathological fear, it is speculated that they may be, to some extent, fear-circuitry-related disorders [6]. Therefore, an enhanced understanding of learned fear is important for the physiological processes underlying these disorders [1,3,4,7,8,9,10].

Learned fear can be experimentally studied using the Pavlovian fear conditioning paradigm. It consists of pairing an emotionally neutral stimulus (a discrete cue, such as a tone, a light, or an odor) with an aversive stimulus (the unconditioned stimulus, US, such as a mild electric shock). As a result of this pairing, the initially neutral stimulus (now being a conditioned stimulus, CS) acquires the ability to elicit a typical behavioral conditioned fear response, such as freezing behavior (immobility with the exception of breathing movements) or fear-potentiated startle reflex (an increase in the amplitude of an acoustically elicited startle response) in rodents (often also measured in humans), or skin conductance response in humans [11,12,13,14]. In this procedure, the US is also associated with the environment (context) in which the US is presented. Thus, the conditioned fear response will be exhibited in both the CS (cued fear conditioning) and the context (contextual fear conditioning) during the subsequent re-exposure to the CS or context [12,13,15]. The association phase between CS and US stimuli constitutes the acquisition phase of fear conditioning, and it is followed by the consolidation phase, during which the memory is stabilized into a consolidated memory (long-term memory) that can be retrieved as needed [16,17]. However, when a mnemonic trace is recalled, it enters a labile state and can be more easily modified or even erased [18]. Indeed, prolonged or repeated re-exposures to the CS determine a gradual weakening of the memory, a process known as extinction [19,20,21].

Conditioned fear response extinction represents an important mechanism in the treatment of fear and anxiety disorders; in fact, exposure therapy, an often used treatment, is conceptually based upon fear extinction [22,23,24]. During exposure therapy, subjects are exposed to stimuli related to the traumatic experience until they suppress their inadequate responses to fear. However, since extinction results in a new mnemonic trace that inhibits the expression of the initial memory, maladaptive defensive behaviors can reappear. This potential for the recovery of maladaptive memory highlights the need to discover more persistent and more robust techniques to decrease maladaptive behaviors [25,26,27]. In this context, pharmacotherapy can be used to increase the efficacy of exposure therapy. Indeed, some drugs, such as D-cycloserine, 3,4-dihydroxy-L-phenylalanine (L-DOPA), propranolol, and serotonin selective reuptake inhibitors (SSRIs), have been shown to enhance fear extinction in animals, and also in translational studies, as well as in humans [27,28]. Recently, it has been reported that administration of acetazolamide, a carbonic anhydrases inhibitor, dose-dependently impaired the consolidation of fear extinction memory of rats trained in contextual fear conditioning, whereas D-phenylalanine, a carbonic anhydrases activator, displayed an opposite action [29]. These findings reveal that the engagement of carbonic anhydrases is essential for providing the brain with the resilience necessary to ensure the consolidation of extinction of emotionally salient events, thus opening the road for novel drug targets.

A series of recent observations are shedding light on the potential involvement of the oxytocinergic system on cognition and, particularly, in the potential use of oxytocin (OXT) as a pharmacological approach to improve exposure-based therapy [30,31]. OXT, a hypothalamic peptide hormone, is well known for its regulatory role of mammalian reproductive processes, such as uterine contraction and milk ejection, but it also plays an important role as a neuropeptide in the brain affecting a wide array of social behaviors, such as pair bonding, social recognition, and maternal care. OXT is involved in the regulation of anxiety and fear, and, interestingly, several neural structures involved in fear extinction express OXT-specific receptors [32,33,34]. These findings, as well as future challenges and perspectives in the field, will be discussed in this review.

## 2. The Central Oxytocinergic System Organization and Potential Therapeutic Applications

OXT is a nine-amino-acid neuropeptide synthesized in the central nervous system and some peripheral tissues (e.g., myenteric and submucous ganglia along the entire human gastrointestinal tract [35], as well as cells and tissues of the reproductive system [36]). However, the central and peripheral release patterns have different temporal dynamics, and it is unclear whether they match and synergize [37].

In the brain of vertebrates, OXT is produced in the magnocellular neurons of the paraventricular (PVN), supraoptic (SON), and accessory (AN) nuclei of the hypothalamus [38]. PVN and SON axonal projections extend through the median eminence (ME) and innervate the posterior pituitary, where OXT is released into the circulation [39]. Magnocellular neurons also extensively project to various forebrain regions, including the prefrontal cortex, anterior olfactory nucleus, nucleus accumbens, lateral septum, hippocampus, and medial and central amygdala [37,40,41,42,43]. Parvocellular hypothalamic and extrahypothalamic PVN projections were found throughout the brain. In particular, they innervate the arcuate nucleus (ARC), the bed nucleus of the stria terminalis (BNST), the ventral tegmental area (VTA), the nucleus of the solitary tract (NTS), the spinal cord, and the hippocampus [44]. Since various subgroups of OXT neurons may innervate distinct brain regions [45], it can be hypothesized that certain stimuli selectively activate neuronal populations with specific intracerebral projections. Indeed, it was recently shown by genetic labeling of OXT neurons activated during fear expression in rats that these “fear-sensitive” OXT cells almost exclusively project to the central nucleus of amygdala, one of the major regions of fear response [46].

OXT is synthesized as a large inactive precursor protein, along with its carrier protein neurophysin I. This precursor is packaged into neurosecretory vesicles and transported axonally to the nerve endings in the neurohypophysis [47]. While it is being transported, the inactive precursor protein is progressively transformed by a variety of post-translational processing steps and hydrolyzed into smaller fragments via a series of enzymes. The last reaction, catalyzed by the peptidylglycine α-amidating monooxygenase, releases the biologically active hormone [48]. OXT stored in large dense-core vesicles present at the nerve endings, soma, dendrites, and axonal varicosities is released by calcium-dependent exocytosis. Suitable stimuli for OXT secretion include labor and infant suckling, as well as sexual stimulation, stressors, and gastric distension [49]. Multiple aminopeptidase enzymes are responsible for OXT degradation. The steady state of mature OXT is controlled by oxytocinase enzyme (cystinyl aminopeptidase), which inactivates OXT via hydrolysis [50,51].

OXT exerts its effects, activating biological macromolecules. To date, only one specific OXT receptor (OXTR) is described. The OXTR belongs to the class-A/rhodopsin GPCR family, in which seven transmembrane-spanning helices connected by three extracellular loops and three intracellular loops are clustered in a bundle [52]. OXTRs are functionally coupled to Gq/11, a class of GTP binding proteins that stimulate, together with Gβγ, the activity of phospholipase C. Due to the high structural homology with the neuropeptide vasopressin (AVP), OXT is able activate the AVP receptor subtypes (V1a, V1b, and V2), although with a lower efficiency [53,54,55]. OXT also readily binds other receptors, such as the transient receptor potential vanilloid-1 receptor (TRPV1) [56,57] or the μ-opioid receptor [58].

Although OXT is best known for its action on the mammary glands and uterus during lactation and childbirth, it is also involved in the neuromodulation of a broad variety of behavioral functions related and unrelated to social behavior. In particular, sexual behavior, maternal care, aggression, pair bonding, and social memory are modulated by brain OXT.

The multiple and known effects of OXT, including its role in controlling stress, anxiety [59,60,61], and modulating fear responses [46,62,63], suggest OXT as an attractive treatment option in human diseases associated with socio-emotional dysfunctions, such as PTSD, generalized anxiety, phobias, and major depressive disorders. Indeed, intranasal administration of synthetic OXT in healthy subjects reduces anxiety levels and promotes various aspects of human social behaviors [64,65]. The same was shown in patients with generalized anxiety, where intranasal administration of OXT produces changes in the activity of some brain areas involved in the regulation of anxiety, such as a decrease in amygdala reactivity during the processing of fearful faces [66,67,68], reduction in the increased activity to sad faces in the mPFC and anterior cingulate cortex [69], and enhancement of amygdala–mPFC and amygdala–anterior cingulate cortex connectivity [70]. Moreover, in PTSD patents, intranasal administration of OXT reduces symptom development, including intrusive re-experiencing [71,72], and enhances functional amygdala–mPFC connectivity, along with inhibitory effects on the amygdala per se [73]. Thus, several studies emphasize OXT as a very interesting add-on therapy for various psychiatric diseases. However, further research is needed to establish OXT as a safe treatment option.

## 3. Fear Memory Extinction and Underpinning Network

Fear extinction occurs when the contingent relationship between the CS and US is compromised following repeated CS presentation without the aversive US. It is characterized by a gradual decrement in the magnitude and frequency of fear responses. It is important to note that extinction does not directly modify the original fear memory. Instead, it leads to the formation of a new inhibitory association CS−no aversive US that competes with the original CS−US memory trace. Thus, extinction does not erase the original association, but rather it implies a new learning that inhibits the original memory from being expressed. In keeping with this view, phenomena such as reinstatement (reappearance of an extinguished fear memory following exposure to unsignaled US after extinction training), spontaneous recovery (reappearance of an extinguished fear memory due to time elapsing after extinction), and renewal (return of an extinguished fear memory in a different context from that in which extinction training took place) after extinction [74] confirm that the original memory is not erased, but remains encoded in the brain without being expressed [75,76,77]. Extinction is context-dependent and this property is exemplified by the renewal effect. For example, if an extinguished CS is presented in the context in which extinction training occurred, fear is suppressed, but, if the CS is presented in a novel context, fear of the CS returns. Thus, the context in which the CS is presented determines which association (CS−US or CS−no US) is retrieved, thereby determining whether fear is expressed or not. Because extinction is a new learning, it also follows the three classical phases of the mnemonic process: acquisition, consolidation, and retrieval [75,76,77].

Emerging evidence from both animal (Table 1) and human (Table 2) studies indicates that fear extinction memory is vulnerable to stress [78,79,80]. Stressors cause impairments in extinction acquisition or retrieval, sometimes in association with increased fear [81]. Moreover, abnormalities in fear extinction are also observed in rodents subjected to stress models, such as “single prolonged stress” (SPS) [82]. In this procedure, animals receive several stressors (restraint, forced swim, and ether anesthesia) in a single session, followed by a 1 week period of rest [82]. These animals exhibit increased anxiety-like behavior in the elevated plus maze, increased contextual fear, and enhanced negative feedback of the HPA axis [82,83]. Moreover, they are characterized by reduced expression of OXTR in mPFC, amygdala, and, to a lesser extent, in the hippocampus [84].

Extinction learning requires the plasticity of a scattered network of brain structures to modify the dynamics of distinct neuronal circuits in a way that a previously learned conditioned response is no longer expressed. Important progress has been made in disclosing in rodents the specific roles played by various structures involved in fear extinction. There is much evidence suggesting that the amygdala complex nuclei (including basal (BA), central (CeA, consisting of the lateral (CeL) and medial (CeM) regions), and the intercalated cell masses (ITCs)) are critically involved in acquisition and storage of memory extinction [85,86,87,88,89,90]. Extinction memory consolidation is mediated by the prelimbic (PL) and infralimbic (IL) areas of the medial prefrontal cortex (mPFC) [91]. The hippocampus has a crucial role in the context-dependent expression of extinction [92,93]. A functional coupling among these areas during fear extinction has been demonstrated in both rodents and humans [94].

Local inactivation of the BA subnucleus with muscimol (a selective agonist of GABA-A receptor) blocks extinction acquisition [95]. Moreover, extinction training induces the immediate early gene c-Fos expression, a marker of neuronal activation, in the BA [96], which, instead, is compromised in animal models with impaired extinction learning [97]. Interestingly, in this nucleus, two distinct populations of projection neurons, whose activity is oppositely correlated with fear conditioning and extinction, were identified: fear neurons and extinction neurons [95]. The first respond to the CS increasing their firing rate during fear memory retention, whereas the second population is activated during retention of fear memory extinction. Thus, extinction training could induce a switch in the balance of CS-evoked activity between these two distinct populations of BA projection neurons [95]. Fear and extinction neurons of the BA exhibit specific reciprocal projections to the mPFC and hippocampus. Specifically, fear neurons are connected with the ventral hippocampus and the PL, whereas extinction neurons are reciprocally connected with IL [98]. These different projections may modulate the balance of BA neuronal activity, allowing expression or not of fear responses. This modulation might be due to an increased GABAergic transmission. Indeed, the gephyrin protein (a structural protein of GABAergic synapse) and mRNAs of other GABAergic markers (such as GABA-synthesizing enzymes) are upregulated after fear extinction training in the basolateral amygdala (BLA) [99,100]. It has been proposed that BA neuronal activity is modulated by specific GABAergic interneurons subtypes, specifically cholecystokinin (CCK^+^) and the cannabinoid receptor type 1 expressing cells [99,101,102]. During extinction, these interneurons would reduce inhibition of extinction neurons which, consequently, increase their responsiveness to the CS [103].

During extinction training, the decrease in CS-elicited fear response parallels with the reduction in CeM neuronal firing [104]. Such effects seem to be related to an inhibitory circuitry, maybe involving BLA and/or CeL, since decreasing the efficacy of intra-amygdala GABAergic transmission impairs extinction memory retrieval [105], whereas enhancing GABAergic transmission facilitates extinction acquisition [106]. The reduced activity of the CeM neurons during extinction could also involve the ITCs. They are clusters of GABAergic neurons located in the external capsule (lateral ITCs, lITCs), lateral to the BLA, and in the intermediate capsule (medial ITCs, mITCs), at the interface of the BLA and CeA. The mITCs are further divided into another two clusters, one located in the proximity of CeL (dorsal mITCs) and another in the proximity of CeM (ventral mITCs). The former project both to CeL and ventral mITCs, whereas the latter project to CeM. Together, these mITC clusters constitute a cellular substrate for gating information flow between the BLA and CeA. They provide feed-forward inhibition to the amygdala: lITCs inhibit the BLA, whereas mITCs inhibit the CeA. These GABAergic neurons are involved in fear extinction. Indeed, selective ablation of mITCs performed after extinction training induces spontaneous recovery of fear responses [107]. On the other hand, extinction increases expression of immediate early genes in mITCs [107,108,109]. Moreover, extinction leads to a potentiation of excitatory BA extinction neuron inputs to ventral mITCs, which, in turn, increase inhibition of CeM output cells [110].

Finally, the reduced activation of CeM cells by BA neurons appears to also be dependent upon excitation of a specific neuronal population of the CeL. In fact, CeM output neurons are under inhibitory control originating in CeL [111,112]. In this amygdaloid nucleus, two distinct populations of neurons have been found, one exhibiting inhibitory (CeL-OFF) and the other excitatory (CeL-ON) responses to the CS after fear conditioning [104,113]. The CeL-OFF neurons express OXTRs [113,114] and exert a tonic inhibitory influence on CeM neurons. For example, release of endogenous OXT in CeL attenuates conditioned freezing [40], probably through the activation of CeL-OFF cells. Moreover, it has been reported that c-Fos expression is increased in these CeL cells in response to contextual fear extinction [115]. This agrees with the proposed function of these neurons in inhibiting the conditioned freezing response [114]. However, further analysis of the activity patterns and recruitment of different CeL neurons is necessary in order to delineate their individual contribution to extinction memory. Overall, these data suggest that the modulation of GABAergic microcircuits within the BLA and CeA is critically involved in the fear extinction.

Experimental results have shown that mPFC is able to exert a dual control over fear expression through separate pathways, each with access to separate sets of inputs and outputs [116]. Whereas PL is involved in the production of fear responses and its inactivation reduces expression of contextual and auditory fear conditioning [86,117], IL is a critical site of plasticity for the inhibition of fear responses and, therefore, for the extinction. These findings have been supported by the observation that PL neuronal activity is critical for fear expression and increases in rats that fail to retrieve extinction memory [118], whereas it is reduced by drugs that decrease fear expression, such as propranolol and cannabidiol [119,120]. Moreover, PL neurons show increased Fos expression after retrieval [108,121]. It was also speculated that PL modulates fear expression through projections to the BA [122], as PL stimulation excites BA neurons [123]. PL interneurons as well seem to play an essential role in the modulation of fear memory. Reduced activity of PL parvalbumin-positive interneurons disinhibits principal neuron output to the BLA and increases conditioned fear [124]. Contrary to PL, IL is involved in fear extinction. Lesions of this area fail to affect within-session extinction (i.e., the decrement in the fear response measured during extinction training), but impair extinction retrieval. This has been confirmed by studies using single-cell recording, showing that IL neurons respond to signal tones during extinction retrieval, and the magnitude of the response is inversely correlated with behavioral outcome [125]. These findings support a specific role of IL in the retrieval of fear extinction. However, this role is debated. In fact, Do-Monte et al. (2015) have reported that optogenetic silencing of glutamatergic IL neurons during auditory fear extinction retrieval does not abolish retrieval, which, instead, is impaired by silencing IL neurons during extinction acquisition. The authors conclude that the IL activity is not necessary for the retrieval of auditory fear extinction [126]. On the other hand, Laurent and Westbrook (2009) have shown that post-extinction training intra-IL infusion of muscimol impairs extinction of contextual fear [86], whereas infusion of picrotoxin (a GABAergic antagonist) facilitates extinction [127,128], supporting a role for this area of mPFC in extinction consolidation. Finally, during extinction, IL neuron excitability increases [129]. In fact, following extinction, IL neurons respond to intracellular application of current by increasing their bursting. This suggests that these neurons are more responsive to their inputs following extinction [129]. Thus, extinction potentiates inhibitory circuits. Functional neuroimaging studies of healthy humans have reported vmPFC (homologue of the rodent IL) activation during extinction [130,131,132,133], and its later recall [130,134]. Skin conductance measures of extinction memory are positively correlated with vmPFC activation [130,134] and vmPFC cortical thickness [135].

Although experimental evidence has shown distinct functions for PL and IL, there is probably some overlapping. Using cell-specific retrograde tracers coupled with optogenetic stimulation, a glutamatergic projection from PL to IL has recently been identified, and its optical stimulation enhances fear extinction [136]. Nevertheless, it is likely that the opposite influence of the IL and PL on fear responses depends, in part, upon the different connections with the amygdala.

The interaction between IL and amygdala is crucial to fear extinction. Specifically, during extinction, the IL controls the signal flow in the BA–CeA circuits, resulting in CeM output neuron inhibition [137]. The IL control of BLA–CeA circuit may be realized through several pathways. IL may activate ventral mITC cells, which, in turn, inhibit the CeM neurons projecting to the effector structures of fear responses [138,139,140]. In addition, or alternatively, IL inputs may synapse on specific populations of BA interneurons, which induce activation of BA extinction neurons and inhibition of BA fear neurons [103,141]. In turn, extinction neurons may influence CeA activity, acting on either inhibitory ventral mITC neurons or CeL neuronal populations that dampen CeM neurons activity, thus reducing fear responses.

The hippocampus plays a dual role in fear extinction: it modulates both extinction acquisition and context dependence of extinction. These hippocampal roles seem to be controlled by direct and indirect projections to the BLA, PL, and IL [142,143].

Pre-extinction muscimol-induced inactivation of the dorsal or ventral hippocampus attenuates or impairs, respectively, fear extinction retention [87,144,145], whereas the same treatment performed immediately after extinction training has no effect on extinction memory [87,146]. Likely, these effects are due to the altered communication between the hippocampus and the amygdala.

Pre-extinction retrieval inactivation of the dorsal and ventral hippocampus also abolishes fear renewal [144,147,148,149]. By contrast, when the same treatment is performed before extinction training, fear renewal occurs [144]. Knapska and Maren (2009) have reported that, during both renewal and extinction memory retrieval, hippocampal c-Fos expression is elevated [108]. Together, these results confirm the role of the hippocampus in the dependence of extinction retrieval on contextual cues. The hippocampus could trigger the context-dependent renewal of conditioned fear responses through its direct projections to the amygdala; both the ventral subiculum and ventral CA1 region of the hippocampus project to the amygdala, although the ventral subiculum projects to BLA and CeM, whereas ventral CA1 projections only terminate in the BA fear neurons [95]. The hippocampus can also affect the amygdala indirectly via its projections to mPFC; the same hippocampal regions that project directly to the amygdala also send projections to the mPFC, both PL and IL [150]. Using cellular imaging c-Fos, Orsini et al. (2011) have examined how the hippocampus and mPFC interact with the BA during fear renewal, showing that BA-projecting neurons within the PL and hippocampus are selectively recruited during the renewal of conditioned fear response. On the contrary, BA-projecting neurons within IL are engaged during extinction recall [142]. This suggests that, during renewal, both the hippocampus and PL actively communicate with the BA.

Extinction-mediating circuits show anatomical and functional similarities in rodents and humans [27,94]. Moreover, in humans, vmPFC exerts an inhibitory influence over the amygdala [151]. Furthermore, studies in healthy subjects show that context-dependent vmPFC–hippocampus activation correlates with extinction success [130,132]. Thus, vmPFC regulation of amygdalar output and vmPFC–hippocampus connections may be a common circuit underlying fear extinction that may be conserved across species. On the other hand, current data imply dysregulation of hippocampus–prefrontal–amygdala circuits in PTSD, characterized by overactivity of brain regions generating fear and a difficulty engaging circuits normally involved in the inhibition of conditioned fear [79].

Animal and human studies demonstrate that stress induces structural and functional changes in extinction circuits. Extinction-impaired stressed animals exhibit amygdala hyperexcitability, coupled with loss of input sources from hippocampal and prefrontal regions. In the amygdala, specifically in the BLA, chronic stress induces dendritic hypertrophy of principal neurons [152,153,154], whereas acute stress causes dendritic retraction [155,156]. However, both acute and chronic stress increase BLA spinogenesis [152,156], which is accompanied by increased neuronal excitability and decreased synaptic inhibition [157,158,159]. On the contrary, stress leads to dendritic retraction in the mPFC and hippocampal neurons [160]. In the mPFC, these alterations have been observed in the IL and are associated with a hypoactivation of this region [160,161,162,163]. Maroun and colleagues (2013) have shown stress-induced plasticity changes in the mPFC–BLA pathway and several molecular abnormalities in the mPFC [156]. Moreover, the hippocampus and hippocampus–mPFC connections show functional downregulation to the stress. For instance, postnatal footshock stress reduces extinction-related ERK phosphorylation in the hippocampal CA1 and long-term potentiation in the hippocampus–mPFC pathway [164,165]. Moreover, extinction deficits produced by chronic stress are accompanied by a decreased hippocampus–PL transmission [166].

## 4. Oxytocin Effects on Fear Memory Extinction

Growing understanding of the neural circuitry subserving inhibitory learning may offer targets for the development of novel drugs that can be used in combination with behavioral training to strengthen fear extinction, thus contributing to the decrease in patients suffering. Several techniques (RNA in situ hybridization, autoradiography, transgenic methods combined with viral expression systems and specific OXTR antibodies) [33,167,168,169,170,171] have been used to learn the brain structures critically involved in fear memory processing [80,172,173,174]. Moreover, OXT has shown anxiolytic properties in animal [175,176,177] and human studies [178,179]. In the following sections we summarize the reports regarding the effects of OXT in the modulation of fear memory extinction, both in animals (see Table 1) and humans (see Table 2).

### 4.1. Preclinical Studies

Data emerging from animal studies show that OXT exerts different effects on fear extinction, depending on the timing and the brain site in which it is administered. The i.c.v. injection of synthetic OXT or a selective OXTR antagonist (OXTR-A) (des Gly-NH2,d(CH2)5(Tyr[Me]2,Thr4)OVT) before auditory fear conditioning session did not affect either fear memory acquisition or recall [180]. However, the OXT-treated rats (0.1 and 1.0 µg) exhibited reduced freezing responses in comparison to controls during subsequent recalling sessions, indicating a facilitated extinction acquisition. In contrast, OXTR-A injection (0.75 µg) caused fear extinction impairment. The OXT-induced facilitatory effect was completely abolished by OXTR-A coadministration. When the compounds were administered before extinction training, contrasting results were found. In rats, OXT treatment at both doses impaired fear extinction, while OXTR-A treatment alone had no effects, but it prevented OXT-induced impairment. In mice, pre-extinction training, OXT injection had an evident dose-dependent effect, since impairment or facilitation of fear extinction were observed following the administration of 0.1 µg or 0.5 µg OXT [180]. Thus, these results indicate that OXT effects on fear extinction are time- and dose-dependent and may be bidirectional. Recently, OXT biphasic effect has also been shown on social motivation in hamsters [181]. According to the authors, both in males and in females, an inverted U-shaped relationship exists between the duration of social interaction and social reward, mediated by OXT within the VTA [181]. However, there is a sex difference in this relationship, and a particular dose of OXT can induce different patterns of behavior in males and females. For example, injection of 9 μL OXT into the VTA decreases social reward in females but increased it in males [181].

Eskandarian et al. (2013) showed that systemic (i.p.) administration of different OXT doses (1, 10, 100, or 1000 µg/Kg) immediately after each extinction training session for four consecutive days slowed down both within-session and long-term contextual fear extinction. The rats receiving doses of 10, 100, or 1000 µg/Kg of OXT exhibited higher freezing levels than saline-treated controls during both subsequent four extinction sessions and a retention test performed seven days after the last extinction training session [182]. Using the same protocol, the authors also investigated the effect of i.p. OXT administration on contextual fear extinction in an animal model of stress-induced extinction impairment (SPS procedure). In SPS rats, OXT did not affect contextual fear extinction [182]. In contrast, Wang et al. (2018) showed that intranasal OXT administration (1 μg) before a cued fear conditioning extinction test amended the SPS-induced fear extinction impairment [183], suggesting that the augmented OXT levels can enhance fear extinction ability [73,184]. The different results in the same animal model may depend on both the different administration time (post-extinction training vs. preconditioning) and the different test (contextual vs. cued fear conditioning). According to the authors, OXT might modulate extinction-process-inhibiting glucocorticoid release. Indeed, it has been reported that, when given before extinction, OXT delays fear conditioning extinction in rats in a bimodal manner, depending on the levels of corticosterone released [185]: decreased corticosterone levels block fear extinction [186,187], whereas enhanced corticosterone levels facilitate fear extinction [187].

The time dependence of OXT effects is also highlighted in the neural structures mediating fear extinction, specifically in the BLA, CeA [33,111,188,189,190,191,192,193], and mPFC [188], which receive oxytocinergic fibers originating from hypothalamic nuclei [40]. OXT-expressing fibers are also found in other memory-related areas, such as the ventral hippocampus [174], in which they play a crucial role on social memory consolidation [174]; however, no studies have investigated the hippocampal OXT role in fear extinction.

In the amygdalar complex, changes in OXT signaling can affect fear extinction in various ways, depending on the interested subnucleus.

Intra-BLA synthetic OXT injection (0.01 µg,) after contextual fear memory retrieval (post-extinction training) impaired extinction in rats. On the other hand, the infusion of the selective OXT agonists WAY-267464 (a full agonist with weak affinity for the OXTR) [194], or (Thr4,Gly7)-oxytocin (TGOT, a specific and potent OXTR agonist) at the same time point resulted in different outcomes: TGOT (7 ng) did not affected fear extinction, whereas WAY-267464 (3 µg) facilitated fear extinction [188]. When these drugs were given before a fear conditioning session, higher freezing levels were observed in the drug-treated than in the vehicle group during retrieval tests, indicating enhanced contextual fear acquisition and impaired extinction [188]. The different effects of synthetic OXT and Way-267464 might be partly due to their interaction with vasopressin type V1A receptors. Exogenous OXT is a potent agonist of this receptor [194,195], whereas WAY-267464 is a high-affinity antagonist of V1A receptors [194,195]. Thus, the OXT at the dose used (0.01 µg) could activate vasopressin receptors in addition to the activation of OXTRs. On the other hand, the WAY-267464 effects could be mediated through antagonizing the V1A [188].

In a different study, pre-extinction training administration of synthetic OXT (0.6, 3, 15, and 75 ng) into the BLA facilitated both within-session and long-term contextual fear extinction [189]. However, whereas the effect on within-session extinction seemed to be dose-independent, that on long-term extinction was dose-dependent. Rats trained under higher doses of OXT (3, 15, and 75 ng) exhibited lower freezing levels than rats trained under the lowest dose (0.6 ng) during the retention test [189]. OXT injection immediately post-extinction training into the BLA did not alter animals’ behavior, suggesting that OXT-related effects were not due to a potentiation of the extinction-consolidation phase. The administration of OXTR-A (OXTR antagonist, 15 ng) directly into the BLA had no effect on conditioned freezing response, but it fully prevented the facilitatory effect when co-administered with OXT [189]. Although the expression of OXTRs on BLA neurons has not been demonstrated [95], it can be speculated that the pro-extinction OXT effects in BLA might be due to the neuropeptide action on BLA extinction and fear neurons activating the former and inhibiting the latter. OXT might act directly on the principal neurons or act on specific classes of BLA interneurons, thus reducing activation of intrinsic amygdaloid pathways by CS [107,196]. Future studies are required to clarify the specific OXT actions on this circuit.

The OXT effects on fear extinction were also studied in rodent models of impaired extinction. For example, the murine strain 129S1SvImJ (S1) shows extinction deficits and several abnormalities in the amygdala and mPFC, such as BLA, CeA, and IL hypoactivation and CeM hyperactivation [197,198,199]. Pre-extinction training intra-BLA OXT (0.01 µg) injection in these mice did not affect either extinction acquisition or retrieval [191]. On the contrary, the same OXT dose applied into the CeA before extinction training facilitated extinction, possibly by promoting the consolidation process. Instead, a 1.0 µg dose of OXT given into the CeA led to an impairment of extinction retrieval, further demonstrating that neuropeptide effects are dose-dependent. These results suggest that the proextinction effects of OXT are specific to the CeA within the amygdala and confirm the observation of previous studies. Using autoradiography of rat brain sections, Huber et al. (2005) observed that OXTRs are specifically located in the CeL. Subsequently, recording spontaneous spiking activity in acute slices of CeA and manipulating with different OXTR agonists and antagonists, they described two neuronal populations: one was excited by OXT receptor activation, whereas another was inhibited by OXT activation. Finally, they found that TGOT application activated a specific GABAergic neuronal population in the CeL that projects to the CeM, enhancing the inhibitory postsynaptic currents. This effect was abolished by application of OXTR antagonists. Thus, OXT enhances excitability of neuronal populations in the CeL, which leads to increased release of GABA in the CeM [111].

These results have been confirmed by further studies, which have shown that OXT administration in the CeA reduces expression of fear in rodents, facilitating fear extinction [40,190]. Female and male rats received TGOT (7 ng) injection into the CeA before the contextual fear retention test. TGOT-treated rats exhibited reduced freezing levels to the context, indicating facilitated extinction [190]. Moreover, in the same study, in vitro electrophysiological recordings showed that TGOT excited CeL interneurons firing and enhanced inhibitory synaptic transmission in CeM neurons projecting to the periaqueductal gray (PAG), the effector structure of freezing behavior. Thus, according to the authors, TGOT accelerated fear extinction via OXTR-expressing CeL neurons, which inhibited the CeM neurons projecting to the PAG [190]. These results have been confirmed by Knobloch et al. (2012) using an optogenetic approach. Optogenetic stimulation of endogenous OXT release into the CeA before contextual fear retention test induced freezing response reduction in female rats. This effect was abolished by OXTR antagonist (d(CH2)5-Tyr(Me)-(Orn8)-vasotocin, 1 µM) injection into the CeA. In vitro optogenetic stimulation increased both action potential frequency of CeL neurons and inhibitory postsynaptic current frequency in CeM neurons. Both effects were blocked by OXTR antagonist administration [40]. Overall, these studies confirm that OXT activates GABAergic CeL neurons, which, in turn, increase GABArgic inhibition in PAG-projecting CeM output neurons, attenuating contextual freezing responses and facilitating fear extinction [40,190]. Data in agreement with these studies have been reported by Hasan et al. (2019). Using a novel method based on virus-delivered genetic-activity-induced tagging of cell ensembles (vGATE), the authors labeled fear-activated OXT-expressing neurons in the female rat hypothalamus. They observed that PVN and SON OXT neurons were activated during contextual fear conditioning, and a fraction of these neurons was reactivated after re-exposure to the same context on the next day. However, when the same animals were exposed to a different context, a significantly higher number of OXT neurons was activated, indicating that a new OXT neuron population was recruited [46]. These fear-activated OXT neurons projected specifically to the CeL. Their optogenetic activation reduced contextual fear, whereas their silencing impaired context-specific fear extinction [46]. Thus, OXT plays a critical role in the CeA, attenuating contextual fear memory and facilitating fear extinction [40,190]. Therefore, OXT in the amygdala seems to act mainly in the CeA influencing CeL-OFF cells. It has been reported that many CeL-OFF neurons (expressing protein kinase C δ (PKCδ^+^)) express OXTRs [114]. Thus, the enhanced OXT release in the CeL by oxytocinergic fibers originating in the hypothalamic nuclei might activate CeL-OFF neurons, which, in turn, would inhibit the CeM output neurons suppressing conditioned fear responses.

Later studies have reported results confirming the region-dependent function of OXT in the amygdala. Synthetic OXT (10 ng) or WAY-267464 (3 µg) applied to the CeA after the first session of contextual fear retention did not induce any effect on freezing response extinction in male rats [188]. Instead, intra-CeA WAY-267464 or TGOT (7 ng) administration before fear acquisition facilitated the subsequent extinction, whereas synthetic OXT had no effects [188]. In another study [189], injection of several doses of OXT (0.6, 3, 15, and 75 ng) into the CeA before the contextual fear extinction training impaired both within-session and long-term extinction. These effects were dose-independent, as the groups treated with different doses of OXT exhibited similar levels of freezing. The coadministration of OXT (15 ng) and an OXT antagonist (desGly-NH2-d(CH2)5(D-Tyr2,Thr4)OVT, OTA, 75 ng) in CeA blocked OXT effects, whereas infusion of OXTA alone facilitated fear extinction. On the contrary, pre-extinction training infusion of TGOT into the CeA impaired within-session but not long-term extinction [189]. The different results by various studies might be due to sex differences (female vs. male rats) and to the different doses used, as OXT effects on fear extinction are, at least in part, sex-specific and dose-dependent [180]. In addition, the drug dose used might also act on vasopressin receptors (V1A), for which they show different affinity and whose activation has opposite effects [31,111]. Moreover, there are multiple mechanisms by which the OXTRs can be transcriptionally induced and differentially involved in several types of behaviors. For instance, in the ventromedial nucleus of the hypothalamus (VMH), OXTR induction is estrogen-dependent and requires protein kinase C activation, whereas, in the CeA OXTR expression is controlled by dopamine through activation of protein kinase A (PKA) [175]. OXT infused in VMH promotes female sexual behavior but has no effect on anxiety; in contrast, OXT administration in CeA has anxiolytic effects but does not influence female sexual behavior [175]. It may speculate that OXT effects on fear extinction in the CeA are PKA-dependent and activated by dopamine D1 receptors. However, there is no experimental evidence.

The partly controversial findings imply a critical role for OXT signaling in amygdala-based regulation of fear learning. OXT may inhibit but also enhance context and cued fear expression and extinction depending on—the site of action within amygdaloid subregions, —the dose used, —the precise time point of application of OXT or its agonists, and—the sex studied. Thus, increasing local OXT neurotransmission during traumatic events may prevent the formation of fear memories, whereas, in contrast, OXT treatment before fear extinction training may delay and impair cued or context fear extinction.

As described above, the IL subregion of the mPFC is a critical site in fear extinction regulation. Concerning OXT activity in this area, a recent study showed that synthetic OXT (10 ng) or WAY-267464 (3 ng) infusions after the first session of fear retention test in male rats facilitated contextual fear extinction. Indeed, OXT- and WAY-treated rats exhibited lower levels of freezing than controls; however, both experimental groups were not different from each other [188]. Similar results have been obtained using TGOT (7 ng) administered into the IL before the second retention test. In contrast, injection of an OXTR antagonist into the same cerebral site had no effect [200]. The same authors have reported that intra-IL TGOT infusion in adolescent rats had no effect on fear extinction, suggesting that OXT effects on fear extinction are age-dependent. This likely is due to the incomplete development of the neuronal circuits in the central nervous system, especially in mPFC [201]. Moreover, in juvenile animals extinction seems to be based on mechanisms and circuits which display distinct features from the adult [202].

It has been speculated that extinction facilitation induced by OXT in the IL could be due to plasticity enhancement and facilitation of glutamatergic synapses in this area. In fact, potentiation of synaptic transmission in the IL underlies fear extinction consolidation [203,204,205,206] and intra-IL OXT administration is associated with long-lasting potentiation of excitatory postsynaptic currents in IL brain slices [207]. However, studies describing the mechanisms of OXT on synaptic plasticity in cerebral sites involved in fear extinction are still scarce. At the circuit level, the increased release of endogenous OXT from hypothalamic nuclei may activate different inhibitory neuronal populations expressing OXTRs in the cerebral sites underlying fear extinction that collaborate together to diminish conditioned fear responses during retrieval and allow for fear extinction.

OXT mechanisms of action within the neural circuits of fear extinction remain unclear. Experimental evidence suggests that this neuropeptide may interact with several neurotransmitter systems. In particular, the anxiolytic effects of OXT might be mediated via the potentiation of GABAergic transmission. GABA is the main inhibitory neurotransmitter in the brain [208,209]. Its inhibitory effects are mediated by ionotropic GABA-A or metabotropic GABA-B receptors. GABA-A receptors are fast-acting ligand-gated chloride channels. Their activation induces an enhanced chloride conductance across the cell membrane, which reduces the neuron ability to raise an action potential and leads to phasic inhibition of the neuron [210]. Moreover, low concentrations of GABA in the extracellular space can persistently activate extrasynaptic GABA-A receptors and generate a tonic inhibition, rendering the neuron less responsive to excitatory stimuli [211]. GABA-B receptors are coupled via G-proteins to either calcium or potassium channels and produce slow and prolonged inhibitory responses [212].

There are only a few studies on the interactions between central OXT and GABA systems. Central OXT acts directly on extrasynaptic GABA-A receptors [213]. Furthermore, OXTRs are located on GABAergic neurons and interneurons in several brain areas, including the amygdala [40,111] and the mPFC [214]. Experimental results support the hypothesis that OXT exerts a direct excitatory effect on GABAergic neurons in the CeL, resulting in the inhibition of target neurons in the CeM, and may thereby enhance fear responses extinction. Recent work has demonstrated that OXT in the mPFC of male rats attenuates anxiety-related behavior engaging GABAergic neurons, which modulate neuronal activation in the BLA and CeA [215].

Interestingly, it has been shown that OXT synergizes with benzodiazepines to inhibit CeM activity. The benzodiazepines (such as diazepam and lorazepam) are commonly used for anxiety disorder treatment. However, they show several side effects, including dependence and withdrawal symptoms after long-term use [216]. These drugs bind to GABA-A receptors and enhance their functions. Viviani et al. (2010) have found that the coapplication of OXT and diazepam facilitates the inhibitory effects of diazepam in rats of either sex. Thus, stronger anxiolysis can be obtained by combining the two substances. OXT has a different site of action than diazepam. In fact, whereas OXT acts directly on CeL (GABAergic) neurons through OXTRs, diazepam inhibits CeM neurons through GABA-A receptors. This makes possible the therapeutic action of OXT in those circumstances in which chronic use of benzodiazepines leads to tolerance [217]. Similar results have been reported in humans. Using functional ultra-high-field imaging in healthy males, Kreuder et al. (2020) compared the effects of OXT and lorazepam on fear-related activity and connectivity in distinct nuclei of the human amygdala. They found that both OXT and lorazepam reduce responses to fearful faces relative to neutral faces activating GABAergic transmission within the CeA [218]. In addition, OXT significantly increased the functional connectivity between the BLA and the CeA, and between the amygdala and the PFC during processing of fearful faces.

### 4.2. Human Studies

Substantial evidence from animal studies has highlighted OXT implication in extinction learning in humans. For the purpose of corroborating the commonly used exposure therapy, efforts are focused on pharmacological augmentation of extinction learning. Relatively few clinical studies have been conducted on the role of OXT as a pharmacological adjunct for exposure-based therapy (see Table 2).

As previously described for animal studies, OXT effects on fear learning is time dependent. Indeed, in humans also, different outcomes were reached with the OXT administration in different phases of the extinction process. Acheson et al. (2013) studied 44 healthy participants administered with intranasal (IN) OXT or placebo before the conditioned fear extinction training. The authors used a fear protocol consisting of a two-graphical presentation (CS): one (CS+) is paired with a 0.5 s electric shock (US), whereas the CS− is never followed by a shock. After this initial acquisition and the drug administration in the extinction phase, the subjects were presented with the graphical CS+ not paired with a shock, or CS−, and the startle response was measured. The authors observed an inhibition of fear extinction (startle response, measured as the difference between startle magnitude in response to CS+ or CS− and the startle response to noise probe alone) in the OXT condition at the beginning of the extinction training, while a similar fear response was shown in the mid-fear-extinction training between OXT and placebo. Instead, when tested on the late extinction phase (24 hr after treatment), the OXT administered group displayed a greater extinction recall relative to placebo, supporting a facilitative effect of OXT treatment in enhancing consolidation of fear extinction training. The study suggests, overall, a potential effect of OXT in fear extinction recall facilitation [184]. The OXT dual effect on extinction, an initial increase in fear response versus an enhanced extinction learning at the end of the extinction training, was also indicated by a randomized double-blind placebo-controlled study of 62 healthy participants [73]. Specifically, subjects were exposed to a Pavlovian fear conditioning and extinction paradigm with concomitant functional magnetic resonance imaging (fMRI). During the conditioning, a brief electrical shock with an intensity perceived as “highly annoying but not painful” was applied to one hand as US, while the CS was chosen as a social or non-social neutral face representation. Throughout the procedure, the skin conductance responses of the hand were sampled with fMRI scan and subtracted to the baseline conductance value. Repeated measures ANOVA analysis permitted discrimination between OXT vs. placebo effect in response to faces representation. OXT or placebo administration was given at the end of the conditioning and before the extinction, which took place on the same day. As previously shown by Acheson et al. (2013), the latter study also showed an initial increase, followed by a late decrease in fear extinction learning in the OXT group. Despite the same outcomes, the two studies differ for the timing of the extinction process. In the former study, the extinction training was comprehensive of the different phases of Pavlovian memory, including the conditioning, reconsolidation, extinction, and recall, as the evaluation was done over 24 h after the OXT administration, being inclusive of the after-conditioning reconsolidation and after-extinction recall. Instead, in the latter study, the procedure was conducted on the same day, excluding the reconsolidation and recall phases, precluding the deduction of OXT effect on the extinction alone [73]. Both neuroimaging studies have also demonstrated a decrease in amygdala reactivity after OXT treatment, in agreement with the growing literature showing the reduction in amygdala activity throughout extinction following social and non-social aversive stimuli [65]. However, more intriguing is the finding of Eckstein et al. (2015) showing the implication of the dorsal region of the mid-medial PFC following OXT treatment. The authors showed an activation of mid-medial PFC only during initial fear extinction training, but not during the late phase of extinction [73], suggesting an increase in the physiological response at the beginning of the learning phase where the behavioral outcome is an increased fear expression.

Hu et al. (2019) showed in a double-blind placebo-controlled study on 61 healthy subjects a facilitatory effect of OXT in the extinction process, tested in the extinction computer task. Specifically, the skin conductance responsivity was measured in a 3-day experiment. The participants, after the conditioning on day 1 during which the presentation of an image was paired with a mild shock on the wrist (CS+), whereas another picture was not paired with a shock (CS−), were administered OXT or placebo and randomly assigned to the retrieval or non-retrieval group. On day 3, the participants were tested for extinction (image presentation without shock) and reinstatement (unsignaled mild shock without picture presentation). Despite an initial equivalent stimulus discrimination, the OXT retrieval group showed a reduction in stimulus discrimination on the late extinction phase, suggesting that OXT interacts with postretrieval processes rather that blocking consolidation to facilitate extinction [219].

In an attempt to investigate the OXT-induced modulation of brain regions known to be involved in the fear extinction process, Kirsch and colleagues (2005) demonstrated a potent reduction in the amygdala reactivity and amygdalar output pathways to midbrain after IN OXT administration. In this double-blind study, 15 healthy male participants were administered with OXT or placebo intranasally and then subjected to an fMRI and functional connectivity concomitant with the presentation of fearful socially and non-socially relevant stimuli. Each participant was asked to match the previous social stimulus with the paired non-social stimulus. Compared to placebo, OXT administration depressed amygdala activation, in particular, when the social stimulus was presented separately. Moreover, functional connectivity analysis showed a reduced amygdala–upper brainstem/midbrain connectivity during OXT condition, suggesting an OXT combined effect on amygdala and midbrain regions mediating behavioral and autonomic fear responses [65].

Despite the findings reported for healthy subjects in clinical studies, a greater complexity of the OXT effect on extinction is reported when evaluated as a pharmacological adjunct for exposure therapy. As was shown in a double-blind study inclusive of 25 patients with social anxiety disorders (SAD), the OXT administration in combination with exposure therapy improved self-appraisal of speech performance across treatment, but these effects did not improve overall symptoms compared to placebo, as evaluated by self-report measures of SAD symptoms (i.e., social phobia fear and avoidance symptoms, etc.) [220], suggesting an OXT effect specifically on the exposure target rather than causing a generalized reduction in social anxiety symptoms. The OXT efficacy as an enhancer of exposure therapy was also tested in 23 arachnophobic subjects [184]. In the study, OXT delivery before exposure therapy did not significantly affect behavioral measures of fear and tended to decrease therapeutic alliance and response to treatment at follow-up [184]. On the other hand, clinical trials involving PTSD patients have shown a positive effect of OXT treatment when associated to exposure therapy. Koch et al. (2016) reported in an fMRI analysis that OXT has the potential to diminish anxiety and fear expression of the amygdala in PTSD, as tested in 37 PTSD patients versus 40 healthy subjects treated with OXT or placebo. In the clinical trials, a BLA and CeM functional connectivity with salience processing areas was also shown. Under placebo, PTSD patients showed a reduced connectivity between CeM and vmPFC, and between BLA and bilateral dorsal anterior cingulate cortex (dACC). The former was reinstated, while the latter was dampened after OXT administration, suggesting that the OXT potential to modulate anxiety and fear expression of the amygdala in PTSD could be partially due to increased control of the vmPFC over the CeM or via decreased salience processing of the dACC and BLA [68]. The same outcome was reported by Flanagan et al. (2018) employing a randomized, placebo-controlled double-blind study where 17 PTSD patients were treated with intra-nasal OXT before each session of exposure therapy. The patients in the OXT group showed lower PTSD- and depression-associated symptoms during the exposure therapy and a higher working alliance score [221]. An fMRI study in agreement with the previous reports suggested a positive effect of OXT administration early post-trauma in PTSD patients. In this study, a single dose of OXT attenuated amygdala–ventromedial and ventrolateral PFC functional connectivity, whereas a repeated OXT dosage reduced PTSD symptom development in recently trauma-exposed emergency department patients with high acute PTSD symptoms [72], showing potential administration frequency-dependent effects of OXT.

It is remarkable that data regarding the side effects (incidence or severity) were not reported in the above studies.

**Table 1:** Summary of preclinical studies in rodents assessing the OXT system manipulation effects on fear extinction.

Animals	Route or Site of Administration	Drug Administered	Time of Administration	Effect on Fear Extinction	Reference
Male Wistar rats	i.c.v.	OXT (1.0 µg/5 µL)	Preconditioning AFC	+	[180]
Male Wistar rats	i.c.v.	OXTR-A (0.75 µg/5 µL)	Preconditioning AFC	−	[180]
Male Wistar rats	i.c.v.	OXT (0.1 or 1.0 µg/5 µL)	Pre-extinction AFC	−Both doses	[180]
Male Wistar rats	i.c.v.	OXTR-A (0.75 µg/5 µL)	Pre-extinction AFC	No effect	[180]
Male CB1 mice	i.c.v.	OXT (0.1 or 0.5 µg/2 µL)	Pre-extinction AFC	−(0.1 µg/2 µL) +(0.5 µg/2 µL)	[180]
Male Wistar rats	i.p.	OXT (1, 10, 100, or 1000 µg/Kg) (multiple injections)	Post-extinction CFC	−(10, 100 or 1000 µg/Kg)	[182]
Male Wistar rats subjected to SPS procedure	i.p.	OXT (1, 10, 100, or 1000 µg/Kg) (multiple injections)	Postextinction CFC	No effect	[182]
Male Sprague–Dawley rats	IN	OXT (1 µg/ µL)	Pre-extinction test of CDFC	+	[183]
Male Sprague–Dawley rats subjected to SPS procedure	IN	OXT (1 µg/ µL)	Pre-extinction test of CDFC	+	[183]
Male Sprague–Dawley rats	BLA	OXT (0.01 µg/0.5 µL)	Postretrieval CFC	−	[188]
Male Sprague–Dawley rats	BLA	WAY-267464 (3 µg/0.5 µL)	Postretrieval CFC	+	[188]
Male Sprague–Dawley rats	BLA	TGOT (3.5 or 7 ng/0.5 µL)	Postretrieval CFC	No effect both doses	[188,201]
Male Sprague–Dawley rats	BLA	OXT (0.01 µg/0.5 µL)	Preacquisition CFC	-	[188]
Male Sprague–Dawley rats	BLA	WAY-267464 (3 µg/0.5 µL)	Preacquisition CFC	-	[188]
Male Sprague–Dawley rats	BLA	TGOT (7 ng/0.5 µL)	Preacquisition CFC	-	[188]
Male Wistar rats	BLA	OXT (75 ng/0.3 µL)	Preacquisition CFC	+	[189]
Male Wistar rats	BLA	OXT (0.6, 3, 15, or 75 ng/0.3 µL)	Pre-extinction CFC (single injection)	+within-session extinction (all doses) +LT extinction (higher doses)	[189]
Male Wistar rats	BLA	OXTA (3 ng/0.3 µL)	Pre-extinction CFC (single injection)	No effect	[189]
Male Wistar rats	BLA	OXT (3 ng/0.3 µL) + OXTA (15 ng/0.3 µL)	Pre-extinction CFC (single injection)	No effect	[189]
Male Wistar rats	BLA	OXT (3 ng/0.3 µL)	Postextinction CFC (single injection)	No effect	[189]
Male juvenile Sprague–Dawley rats (P27)	BLA	TGOT (3.5 or 7 ng/0.5 µL)	Postretrieval CFC	-Both doses	[201]
Male juvenile Sprague–Dawley rats (P27)	BLA	TGOT (7 ng/0.5 µL)	Preacquisition CFC	No effect	[201]
Male 129SvImJ mice	BLA	OXT (0.001 µg/side)	Pre-extinction AFC (single injection)	No effect	[191]
Male 129SvImJ mice	CeA	OXT (0.01 or 1.0 µg/side)	Pre-extinction AFC (single injection)	+(0.01 or 1.0 µg/side) −(1.0 µg/side)	[191]
Male Sprague–Dawley rats	CeA	TGOT (7ng/0.5 µL)	Pre-retention CFC test	+	[190]
Female Wistar rats	CeA	Endogenous OXT (released following optogenetic stimulation)	Pre-retention CFC test	+	[40]
Female Wistar rats	CeA	OXTA (21 ng/0.5 µL)	Pre-retention CFC test	No effect	[40]
Female Wistar rats	CeA	OXTA + endogenous OXT	Pre-retention CFC test	No effect	[40]
Male Sprague–Dawley rats	CeA	OXT (0.01 µg/0.5 µL)	Preacquisition CFC	No effect	[188]
Male Sprague–Dawley rats	CeA	WAY-267464 (3 µg/0.5 µL)	Preacquisition CFC	+	[188]
Male Sprague–Dawley rats	CeA	TGOT (7 ng/0.5 µL)	Preacquisition CFC	+	[188,201]
Male Sprague–Dawley rats	CeA	OXT (0.01 µg/0.5 µL)	Postretrieval CFC	No effect	[188]
Male Sprague–Dawley rats	CeA	WAY-267464 (3 µg/0.5 µL)	Postretrieval CFC	No effect	[188]
Male Wistar rats	CeA	OXT (75 ng/0.3 µL)	Preacquisition CFC	+	[189]
Male Wistar rats	CeA	OXT (0.6, 3, 15, or 75 ng/0.3 µL)	Pre-extinction CFC	−Both within-session and LT extinction (all doses)	[189]
Male Wistar rats	CeA	TGOT (7 ng/0.3 µL)	Pre-extinction CFC	−Within-session extinction No effect on LT extinction	[189]
Male Wistar rats	CeA	OXTA (15 ng/0.3 µL)	Pre-extinction CFC	+Both within-session and LT extinction	[189]
Male Wistar rats	CeA	OXT (15 ng) + OXTA (75 ng)	Pre-extinction CFC	No effect	[189]
Male Wistar rats	CeA	OXT (3 ng/0.3 µL)	Postextinction CFC	−LT extinction	[189]
Male juvenile Sprague–Dawley rats (P27)	CeA	TGOT (7 ng/0.5 µL)	Preacquisition CFC	−	[201]
Male juvenile Sprague–Dawley rats (P27)	CeA	TGOT (7 ng/0.5 µL)	Postretrieval CFC	No effect	[201]
Male Sprague–Dawley rats	IL-mPFC	OXT (0.01 µg/0.5 µL)	Postretrieval CFC	+	[188]
Male Sprague–Dawley rats	IL-mPFC	WAY-267464 (3 µg/ µL)	Postretrieval CFC	+	[188]
Male Sprague–Dawley rats	IL-mPFC	TGOT (7 ng/0.5 µL)	Pre-extinction CFC	+	[200]
Male Sprague–Dawley rats	IL-mPFC	OXTA (153 µmol/L)	Pre-extinction CFC	No effect	[200]
Male juvenile Spague–Dawley rats (P27)	IL-mPFC	TGOT (7 ng/0.5 µL)	Postretrieval CFC	No effect	[201]

+ Enhancement; − Impairment; AFC, auditory fear conditioning; BLA, amygdala basolateral complex; CeA, central amygdala; CDFC, cue-dependent fear conditioning extinction test; CFC, contextual fear conditioning; i.c.v., intracerebroventricular administration; IL-mPFC, infralimbic area of the medial prefrontal cortex; IN, intra-nasal administration; i.p., intraperitoneal administration; LT, long-term extinction; OXTA, oxytocin antagonist; OXT, synthetic oxytocin; P27, post-natal day 27; SPS, single prolonged stress procedure; TGOT, oxytocin agonist; WAY-267464, oxytocin agonist.

**Table 2:** Summary of clinical studies assessing the OXT system manipulation effects on fear extinction.

Subjects (Number)	Study Design	Route of Administration	Drug Administered	Time of Administration	Effect on Fear Extinction	Reference
Healthy volunteers (44)	Double-blind Placebo-controlled study	IN	OXT (24 IU)	Pre-extinction	−At the beginning of extinction training No effect in the middle phase +in the late phase	[184]
Healthy volunteer (62)	Double-blind Placebo-controlled study	IN	OXT (24 IU)	Post-conditioning and Pre-extinction	−At the beginning of extinction training +in the late phase	[73]
Healthy volunteers (61)	Double-blind Placebo-controlled study	IN	OXT (40 IU)	Post-conditioning	+	[219]
Healthy volunteers (15)	Double-blind Placebo-controlled study	IN	OXT (27 IU)	Pre-conditioning	No effect	[65]
SAD Patients (25)	Double-blind Placebo-controlled study	IN	OXT (24 IU)	Pre-exposure therapy	+(in combination with exposure therapy)	[220]
Arachnophobic Patients (23)	Double-blind Placebo-controlled study	IN	OXT (24 IU)	Pre-exposure therapy	-	[184]
PTSD Patients (37) vs. Healthy Subjects (40)	Placebo-controlled Crossover study	IN	OXT (40 IU)	Pre-exposure therapy	+(in combination with exposure therapy)	[68]
PTSD Patients (17)	Double-blind Placebo-controlled study	IN	OXT (40 IU)	Pre-exposure therapy	−	[30]
PTSD Patients (range = 37–41)	Double-blind Placebo-controlled study	IN	OXT (40 IU- single dose)	Pre-fMRI	−	[72]
PTSD Patients (107)	Double-blind Placebo-controlled study	IN	OXT (40 IU- multiple doses)	Post-trauma	+	[72]

+ Enhancement; − Impairment; IN, intranasal administration; OXT, synthetic oxytocin; fMRI, functional magnetic resonance imaging; IU, international units.

## 5. Challenges and Perspectives on OXT Research and Therapeutic Exploitation

Fear extinction is an adaptive form of learning allowing inhibition of the expression of conditioned fear responses to changed environmental contingencies. Failure of extinction can lead to excessive fear, as in some forms of trauma- and anxiety-related disorders. Such disorders are often treated with exposure therapies conceptually based upon fear extinction. However, these therapies show some limits, as they tend to produce transient fear reduction that is bound to the context in which the therapy is administered. One strategy to improve exposure therapies is to associate them with adjunctive pharmacological agents, such as antidepressants (SSRIs), D-cycloserine, glucocorticoids (cortisol), and α2-adrenoceptor antagonists (yohimbine) [27]. Recent research, both in animal models and human subjects, suggests that OXT has anxiolytic effects and may modulate fear extinction. Accordingly, OXT might be considered as an adjunctive treatment to extinction-based therapies. Here, we reviewed recent studies that addressed this issue, focusing on the OXT effects on supposed cerebral networks underlying fear extinction. OXT modulates fear memory extinction, acting mainly on GABAergic neurons of BLA, CeA, and IL. However, more data are needed to find out how OXT acts on extinction neurocircuits. In fact, there is evidence for both extinction-impairing and extinction-improving effects. Increasing local OXT neurotransmission during traumatic events may prevent the formation of fear memories, whereas OXT treatment before fear extinction training cannot be excluded to even delay and impair fear extinction. Since treatment before extinction training would be the comparable time point for psychotherapy in fear-related disorder patients, caution is needed before recommending OXT for the add-on treatment of these disorders, especially in trauma of a non-social nature. Most human studies have used intranasally administered OXT, a noninvasive route, but nasal sprays are limited in terms of dosage control of dosage, absorption, and drug response [221]. Thus, further animal studies are necessary to examine doses, timing of administration, gender dependence of efficacy, and to develop study the timing of OXT effects. Moreover, the signal transduction pathways regulating OXTR expression and binding in each brain region must be clarified. Finally, a specific analysis of the role of hippocampal OXT in nonsocial fear extinction seems to be necessary.

Evidence reviewed here demonstrates that fear extinction depends on complex networks that include interactions between multiple amygdaloid parallel circuits co-ordinated by hippocampal and mPFC activity. Despite these recent advances, several questions remain. For instance, although interneurons are a small cell population in the mPFC and amygdaloid nuclei, they are critical for inhibition. Thus, defining the specific roles of several interneuron classes in circuits underpinning fear extinction will provide a clearer understanding of these circuits. Moreover, hippocampal–mPFC–amygdala circuits mediate context dependence of fear memories after extinction; however, future studies of context processing in various psychiatric disorders seem necessary. As the neural networks underpinning fear extinction are evolutionarily conserved circuits, a detailed understanding of these circuits will allow the development of more effective approaches to treat the disorders caused by dysfunctions in these circuits.

## Abbreviations

AFC	auditory fear conditioning
AN	accessory nuclei
ARC	arcuate nucleus
AVP	vasopressin
BA	basal amygdala
BLA	basolateral amygdala
BNST	bed nucleus of the stria terminalis
CCK	cholecystokinin
CDFC	cued-dependent fear conditioning
CeA	central amygdala
CeL	central lateral amygdala
CeM	central medial amygdala
CFC	contextual fear conditioning
CS	conditioned stimulus
dACC	dorsal anterior cingulate cortex
fMRI	functional magnetic resonance imaging
HPA	hypothalamus pituitary axis
IL	infralimbic area
IN	intranasal
ITCs	intercalated cell masses
LA	lateral amygdala
ME	median eminence
mPFC	medial prefrontal cortex
NTS	nucleus of the solitary tract
OXT	oxytocin
OXTR	OXT receptor
PAG	periaqueductal gray
PL	prelimbic area
PTSD	post-traumatic stress disorder
PVN	paraventricular nucleus
SAD	social anxiety disorders
SON	supraoptic nucleus
SPS	single prolonged stress
TRPV1	transient receptor potential vanilloid-1 receptor
US	unconditioned stimulus
VMH	ventromedial nucleus of the hypothalamus
VTA	ventral tegmental area
